# Disasters at Mass Gatherings: Lessons from History

**DOI:** 10.1371/currents.RRN1301

**Published:** 2012-03-12

**Authors:** Lee Soomaroo, Virginia Murray

**Affiliations:** ^*^Emergency Specialist Registrar in the UK, and member of the World Health Organisation/Health Protection Agency UK, Collaboration of Mass Gathering Emergency Medicine and ^†^Head of Extreme Events and Health Protection Centre for Radiation, Chemicals and Environmental Hazards, Health Protection Agency, London UK

## Abstract

Introduction

Reviews of mass gathering events have traditionally concentrated on crowd variables that affect the level and type of medical care needed. Crowd disasters at mass gathering events have not been fully researched and this review examines these aiming to provide future suggestions for event organisers, medical resource planners, and emergency services, including local hospital emergency departments.

Methods

A review was conducted using computerised data bases: MEDLINE, The Cochrane Library, HMIC and EMBASE, with Google used to widen the search beyond peer-reviewed publications, to identify grey literature. All peer-review literature articles found containing information pertaining to lessons identified from mass gathering crowd disasters were analysed and reviewed. Disasters occurring in extreme weather events, and environmental leading to participant illness were not included. These articles were read, analysed, abstracted and summarised.

Results

156 articles from literature search were found detailing mass gathering disasters identified from 1971 – 2011. With only 21 cases found within peer-review literature. Twelve events were further documented as a case reports. Five events were examined as review articles while four events underwent commissioned inquiries. Analysis of cases were categorised in to crowd control, event access, fire safety, medical preparedness and emergency response.

Conclusions

Mass gathering events have an enormous potential to place a severe strain on the local health care system, and a mixture of high crowd density, restricted points of access, poor fire safety, minimum crowd control and lack of on-site medical care can lead to problems that end in disaster.

## **Introduction**

Mass gatherings require the provision of medical services for large populations who have assembled under unusual circumstances. Mass gatherings, including scheduled events in sports facilities, air shows, rock concerts, outdoor celebrations, and visits by dignitaries, vary in their complexity and demand for medical services. 

The definition of a mass gathering itself is not without debate. The National Association of EMS Physicians (NAEMSP) defined it as: “Organized emergency health services provided for spectators and participants at events in which at least 1000 persons are gathered at a specific location for a defined period of time [1], while the WHO [2] describes it as “An organised or unplanned event where the number of people attending is sufficient to strain the planning and response resources of the community, state or nation hosting the event”. The United Nations International Strategy for Disaster Reduction (UNISDR) defines a disaster as “a serious disruption of the functioning of a community or a society involving widespread human, material, economic or environmental losses and impacts, which exceeds the ability of the affected community or society to cope using its own resources”. In the context of this review of mass gathering incidents, a disaster is to be considered as a calamitous event occurring suddenly and causing great loss of life or damage to surroundings. 

Historically, peer-reviewed literature has concentrated on crowd variables that affect the level and types of medical need at a mass gathering event [3], studying patient presentation rates of illness and minor injuries, while also providing possible prediction tools for first-aid providers and local hospitals [4]. However there is a lack of available evidence analysing crowd disasters at mass gathering events providing future suggestions for event organisers, medical resource planners, and emergency services, including local hospital emergency departments. 

This review aims to analyse previous cases of disaster at mass gathering events, documenting the lessons identified, to provide considerations when planning for future events. With careful assessment of mass gathering events as a whole it will be possible to plan ahead for the potential number of attendees, consider health and safety aspects of planning for a mass gathering, plan for potential disasters and decrease the risk of the happening, provide a more effective allocation of health resources.

## **Method**

This literature review concentrates on crowd disasters at mass gathering events, focusing on predominantly on case reports and literature reviews citing particular lessons identified from previous disasters.  

  A literature search was carried out using Medline, Cochrane, HMIC and Embase. A free-text search was also conducted using Google to link mass gathering events to disaster incidents in order to widen the search beyond peer-reviewed publications which included grey literature (media reports, unpublished reports and commissioned inquiries). All peer-review literature articles found containing information pertaining to lessons identified from mass gathering crowd disasters were analysed and reviewed. Articles describing disasters occurring in extreme weather events such as heat-related illness, and environmental hazards leading to illness such as disease outbreak were not included. Citations within articles were further searched to identify additional references that would inform this review. 

Keywords: Mass Gatherings, Mass Gathering Medicine, Disasters, Sporting Events, Olympic Games, Festivals, Concerts, Disaster, Stampede, Fire, Terrorism, Religious Gathering, Air Show.

## **Results**

In total, 156 reported mass gathering incidents from 1971 – 2011, were identified through literature search. While some reports provided valuable information regarding mass gathering disaster occurrence, most cases were descriptive rather than analytical, consequently very few literature reports (only 21) were found to inform subjects of lessons identified from disasters at mass gathering events. 17 of these reports had been only reported in journal publications, with four reports having undergone commissioned inquiries. 

 21 reported mass gathering incidents have been read, analysed, abstracted, referenced and complied in chronological order and referenced. Main learning points have been identified and further categorised in to 5 key areas for further discussion:

- Overcrowding and Crowd Control
- Event Access Points
- Fire Safety Measures
- Medical Preparedness
- Emergency Response

**Figure:**
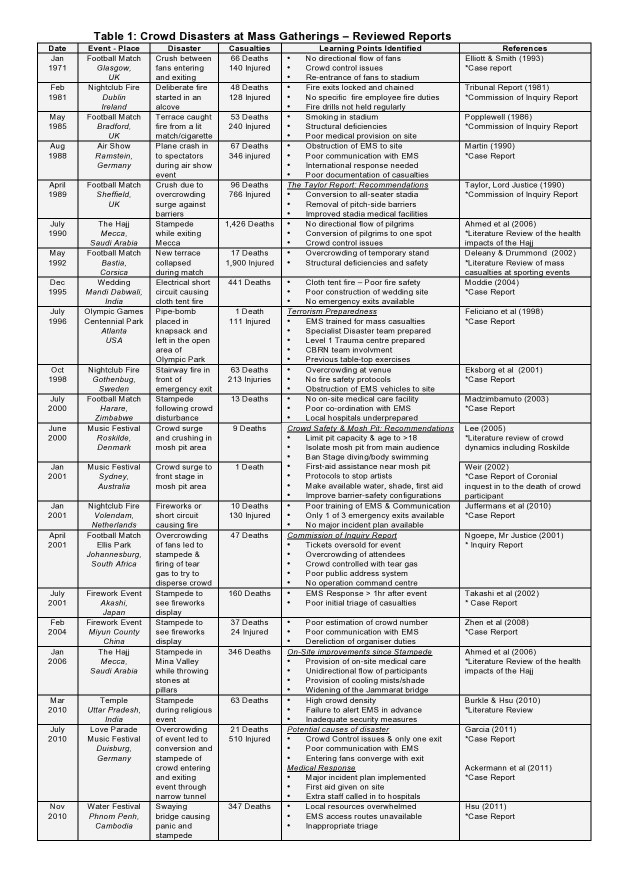
Table 1: Crowd Disasters at Mass Gatherings- Reviewed Reports. 

## Discussion 

Generic themes from disasters at mass gathering events emerge from this collection of literature. If considered carefully in planning for mass gatherings, these might reduce morbidity and mortality should a disaster occur. These themes include overcrowding and crowd control, event access points and fire safety measures. In addition pertinent issues identified in medical prepraredness and emergency responses are also considered. 

### Overcrowding and Crowd Control 

Several studies have highlighted that despite venue capacities being completely full, further crowd members still tried to gain access, either due to overselling of tickets or by people turning up just before or after the start of the event. An adequate ticketing system and public address measures to inform crowds of no further access to an event once capacity is reached is essential. 

 Training of stewards and security staff in crowd control should be implemented before an event in an effort to improve crowd safety and avoid panic should overcrowding occur. During a football match in Ellis Park South Africa, tear gas was thrown in to a crowd by event security in an effort to disperse intense overcrowding. This unfortunately served to incite panic and cause stampede [5]. 

 Overcrowding has been linked to the collapse of a temporary stand at a football match in Corsica [6], while in the UK, the Hillsborough disaster in April 1989 led to a landmark report by Lord Justice Taylor, to change all football stadia in the UK to all-seater venues, improving crowd safety and control as well as removing pitch-side barriers and improving medical facilities on-site [7]. 

 Two cases at music events have stressed the need to provide ‘mosh’ pit safety. Since ‘moshing is a dance in which participants push or slam into each other in enclosed spaces, primarily during live music events, mosh pit crowd safety guidelines have been implemented, which include isolating the area from the main audience, provision of nearby first aid, and protocols to stop artists performing should crushing develop [8]. 

### Event Access Points 

One key structural element to an event venue is the provision of adequate site access, not only for participants but also for emergency medical services. 

The Ibrox stadium disaster in 1971 [9] involved fans leaving the stadium getting caught up with fans entering the stadium at the same time, leading to crush and stampede when all fans heard a goal being scored. Further episodes of crowd convergence have occurred at a stampede causing the death of 1,426 during the Hajj in 1990 where pilgrims spontaneously rushed to leave Mecca via one exit [10], and during the Love Parade in Germany 2010 where overcrowding led to devastating consequences in a tunnel providing the only means of entrance and exit to fans of a music festival [11] [12]. To address this, many mass gathering events now have access points solely for entrance or exit at the site, promoting a unidirectional flow of crowd members and dramatically reducing the risk of crowd convergence. 

Emergency medical services unable to access event sites was found to be a major factor in the prolonged response time to the Ramstein air show disaster, where members of an Italian Air Force display team collided and crashed to the ground [13]. While at a traditional festival in Cambodia, no access routes were available for emergency medical services to a stampede, severely hindering the medical response [14]. It is suggested the planning for future events includes provision of easy, unobstructed access for EMS vehicles.

### Fire Safety Measures 

Fire safety has become an increasing part of emergency planning, however several lessons can still be identified. The stardust fire in Dublin 1981 was thought to have been deliberately started from the ignition of newspapers under flammable seats resulting in 48 deaths and 128 injuries [15].The Bradford City Stadium Disaster is thought to have started by a dropped match or cigarette falling through floorboards to rubbish below [16]. 441 deaths occurred at an Indian wedding due to a synthetic cloth tent catching fire in a large area with no means of emergency exit [17], and 375 attendees were caught in a nightclub fire in Gothenburg, Sweden when the venue was only supposed to hold 150 with the fire starting in front of the emergency exit [18]. Only one of three emergency exits were available during a nightclub fire in Volendam, Netherlands [19]. 

From the five events reviewed it appears that all fire disasters had similar attributes that emergency planners should consider when planning for a mass gathering: 

       Several emergency exits should be made available at any planned event.

- Emergency exits should be free from obstruction, not blocked and functioning properly, with appropriate signage.
- Adherence to fire safety protocols is key including prevention of overcrowding of venues
- Event employees should be allotted specific duties to be performed in the event of the fire, with regular fire drills held on the premises
- Full site fire evacuation plans are essential. These could include signage to evacuation points.

Medical Preparedness 

Provision of on-site physician-level medical care at mass gatherings has been shown to significantly reduce the number of patients requiring transport to hospital and therefore reducing the impact on the local ambulance services [20]. The majority of non-disaster injuries and medical complaints at a mass gathering can be effectively treated on scene, which reduces the number of hospital referrals and patient presentation rates to hospital [21]. 

Following the Hillsborough Stadium disaster 1989, Lord Justice Taylor [22] made suggestions to improve on-site medical care (Box 1): 

**Figure:**
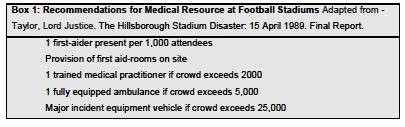
Box 1: Recommendations for Medical Resources at Football Stadiums. 

On-site medical care has also dramatically improved following several stampedes occurring during the annual Islamic pilgrimage of the Hajj [10]. The most notable event in 2006 resulted in 346 deaths at a bottleneck area in the Mina Valley. The provision of specially equipped medical care facilities, helipads, electronic surveillance, shading and cooling mists have helped to reduce crowd morbidity and mortality [10].

- Local hospitals should also be involved with emergency planning in preparation for potential mass gathering disasters. Madzimabuto [23], in a review of a stampede at a football match identified the following hospital preparedness deficiencies identified as:
- Emergency department only first aware when injured arrived
- No major incident plan was prepared which led to the emergency department being overwhelmed
- A hospital command centre was not set up
- Staff reinforcements were unable to be contacted
- Medical teams were not organised to prioritise mass casualty care
- The media arrived, distracting emergency department personnel
- Supporting hospitals were not involved in a timely manner

It is suggested that when planning for a mass gathering event, local hospitals should be involved in healthcare provision not only on-site but also for their own planning of mass casualty events allowing for potential occurrence of disaster.

For mass gatherings on larger scale such as Olympic Games, an assessment of terrorism risk assessment might be necessary, with further medical provisions available during these planned events. For example Feliciano et al [24] reported that there was extensive pre-Olympics preparation (Box) at the regional Level 1 trauma centre before the Atlanta Games in 1996. In particular the pre-hospital training (point 4, box 2) resulted in an excellent hospital-wide response to the multi-casualty event following the explosion of a pipe-bomb in Centennial Park during these 1996 Olympics which resulted in 1 death and 21 injured on scene.

**Figure:**
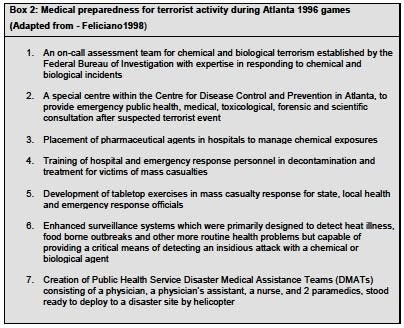
Box 2: Medical preparedness for terrorist activity during Atlanta 1996 games 

### Emergency Response 

Many of the case reports highlighted a poor response time for emergency services but it is unknown if each event reviewed had a mass gathering major incident plan available. However, it is necessary to have one in place to co-ordinate fast and effective emergency response, to potentially improve morbidity and mortality. 

Poor initial communication with emergency medical services was a key finding in not only the Ramstein air show disaster [13], but also in stampedes at the Miyun Country bridge disaster in China [25], the Akashi firework event in Japan [26]  and at a religious temple in Uttar Pradesh [27]. These highlighted poor triage of casualties. Indeed in one instance it was triage was initiated approximately 80 minutes after the incident occurred [26].

It has been suggested that emergency planning has considered that emergency personnel on-site have adequate training and experience in disaster medicine, utilising appropriate triage methods. One analysis of medical care at mass gatherings by Sanders et al [28] suggested:

- Basic first aid within 4 minutes
- Advanced Life Support within 8 minutes
- Evacuation to a medical facility within 30 minutes.

However these suggestions are based on only low patient numbers requiring triage, and arose from low-level evidence and expert judgement, which the authors stressed is in need of further research [28].  

Event preplanning using these principles has been described by [12] in relation to the 2010 Love Parade in Germany when major medical preparations were put in place. They reported on patient care delivery at this mass gathering, where following a stampede, 21 died and over 510 were injured. Once a disaster was called at the site, additional emergency healthcare personnel were brought in, and extra treatment facilities were created to deal with the situation. First-aid posts and emergency treatment and triage stations were set up on site, dealing with a high volume of patients. The formation of trauma teams made it possible to control patient flows appropriately to all hospitals. However, even with such well planned emergency response, adverse consequences occurred which demonstrates again the complexity of planning for such mass gathering events. 

## Summary 

Mass gathering events have a potential to place a severe strain on the local health care system, with a mixture of high crowd density, restricted points of access, limited crowd control and lack of sufficient on-site medical care and emergency response can increase the risk of disaster. 

Many challenges are faced by mass gathering event organisers, medical resource planners, and emergency services, including local hospital emergency departments in order to provide a safe event. Analysis of previous crowd disasters indicates a need for early detailed planning of crowd policies, evacuation procedures and involvement of emergency services which are necessary to promote and provide a successful event. This review highlights several key considerations that could impact on health resources include:

- Preplanning for mass gathering events is key and should include health management and major incident planning.
- Adequate crowd security and emergency medical services need to be provided at a mass gatherings taking account of crowd size and factors such as event type and external environmental conditions
- Emergency Medical Services with adequate training and experience, in the management of multiple medical casualties need to be available. Pre-planning with local hospitals will aid the emergency response.

      Health planning should include

- Training of stewards and security staff in crowd control
- An adequate ticketing system to limit overcrowding
- A functioning and adequate public address system
- Specified entrance and exit points to an event to help ensure a unidirectional flow of crowd

- Evacuation plans including fire safety should be available for all mass gathering events with information passed on to attendees in a clear fashion. Exit routes should be clear and free from obstruction, and plans should be in place for co-ordinated and safe evacuation.
- Every mass gathering event should have a major incident and mass casualty plan which should be activated in the event of a disaster.

Further studies of mass gathering events are warranted to advance not only the epidemiological knowledge base, but also to gain further lessons of emergency preparedness and response. 

##  Funding information 

2 Months out-of-placement training (OOPT) secondment to the Health Protection Agency UK for Dr Soomaroo kindly funded by HPA medical trainees fund. 

##  Acknowledgements 

With thanks to Mark Nunn of the World Health Organisation and Mark Salter at the Health Protection Agency UK. 

##  Competing interests 

The authors have declared that no competing interests exist.

Abbreviations

DMAT – Public Health Service Disaster Medical Assistance Teams; EMS – Emergency Medical Services; NAEMSP – National Association of EMS Physicians; UNISDR – United Nations Strategy for Disaster Reduction 

## References

[ref-2252822345] De Lorenzo RA. Mass gathering medicine: a review. Prehosp Disaster Med 1997;12:68-72 10.1017/s1049023x00037250 10166378

[ref-4175877291] World Health Organisation. Communicable disease alert and response for mass gatherings: key considerations. June 2008

[ref-3261425444] Milsten Am, Maguire BJ, Bissell RA, Seaman KG. Mass-Gathering Medical Care: A Review of the Literature. Prehosp Disast Med 2002;17:151-162 10.1017/s1049023x00000388 12627919

[ref-3117565626] Arbon P, Bridgewater FH, Smith C. Mass gathering medicine: a predictive model for patient presentation and transport rates. Prehosp Disaster Med 2001;16:150-158 10.1017/s1049023x00025905 11875799

[ref-1608665674] Ngoepe Mr Justice B. Final Report Commission of Inquiry into the Ellis Park Stadium Soccer Disaster of 11 April 2001. http://www.info.gov.za/view/DownloadFileAction?id=70241

[ref-3092204573] Deleany JS, Drummond R. Mass casualties and triage at a sporting event. Br J Sports Med 2002;36:85-88 10.1136/bjsm.36.2.85 PMC1724488 11916887

[ref-3017554430] Taylor, Lord Justice. Final Report into the Hillsborough Stadium Disaster, CM 962. London, HMSO. 1990.

[ref-642418363] Weir, I. Findings and Recommendations by the Coronial Inquest into the Death of Jessica Michalik. 2002;1-15 http://www.crowdsafe.com/BDOInquestreportWeirFINAL120802.pdf

[ref-4026453063] Eliott D, Smith D. Football stadia disasters in the united kingdom, learning from tragedy? Organisation Environment 1993;7:205-229

[ref-3274362632] Ahmed QA, Arabi YM, Memish ZA. Health Risks at the Hajj. Lancet 2006;367:1008-1015 10.1016/S0140-6736(06)68429-8 PMC7137144 16564364

[ref-433168844] Garcia LM. Pathological Crowds: Affect and danger in responses to the Love Parade disaster at Duisburg. Dancecult. Journal of Electronic Dance Music Culture. 2011;2;html http://www.dj.dancecult.net/index.php/journal/article/view/66/102

[ref-4167507734] Ackermann O, Lahm A, Pfohl M, Kother B, Lian TK, Kutzerm Weber M, Marx F, Vogel T. Hax P-M. Patient care at the 2010 Love Parade in Duisburg Germany. Dtsch Arztebl int 2011; 108:483-489 10.3238/arztebl.2011.0483 PMC3149288 21814525

[ref-2457488815] Martin TE. The Ramstein Airshow Disaster. J R Army Med Corps 1990;136:19-26 10.1136/jramc-136-01-03 2319499

[ref-3863485166] Hsu, EB. Human Stampede: An Unexamined Threat. Emergency physicians Monthly 2011

[ref-2139442695] Report of the Tribunal of Inquiry on the fire at the Stardust, Artane, Dublin on the 14th February, 1981. by Tribunal of Inquiry on the Fire at the Stardust, Artane, Dublin on the 14th February, 1981 (Ireland) Published in 1982, Published by the Stationary Office (Dublin). http://www.lenus.ie/hse/bitstream/10147/45478/1/7964.pdf

[ref-3913207233] Popplewell, Mr Justice O. Committee of Inquiry into Crowd Safety and Control at Sports Grounds. Final Report. Cmnd 9710 Jan. London, HMSO. 1986

[ref-1239630910] Moddie M. Accidents and Missed Lessons. Frontline 2004;16:13-14

[ref-2197298725] Eksborg A-L, Elinder H, Mansfield J, Sigfridsson S-E, Widlund P. Fire in Gothenburg. 1998 October 29-30. Report RO 2001:02 (o-07/98) by the Swedish Board of Accident Investigation

[ref-3243558144] Juffermans J, Bierens JJLM. Recurrent medical response problems during five recent disasters in the Netherlands. Prehosp Disaster Med 2010 Mar-Apr;25(2):127-36 10.1017/s1049023x00007858 20467991

[ref-3194679412] Grange J, Baumann GW, Vaezazizi R. On-site physicians reduce ambulance transports at mass gatherings. Prehosp Emerg Care 2003;7;322-326 10.1080/10903120390936518 12879381

[ref-2261050515] Olapade-Olaopa EO. Along TO, Amanor-Boadu SD, Sanusi AA, et al. On-site physicians at a major sporting event in Nigeria. Prehosp Disast Med 2005;21:40-44 10.1017/s1049023x00003307 16602264

[ref-70551745] Taylor, Lord Justice. Final Report into the Hillsborough Stadium Disaster, CM 962. London, HMSO. 1990.

[ref-971363322] Madzimbamuto FD. A hospital response to a soccer stadium stampede in Zimbabwe. Emerg Med J. 2003 Nov;6:556-9 10.1136/emj.20.6.556 PMC1726215 14623853

[ref-131670395] Feliciano DV, Anderson GV, Rozucki GS, et al. Management of Casualties from the Bombing at the Centennial Olympics. Am J Surg 1998;176:538-543 10.1016/s0002-9610(98)00263-3 9926786

[ref-153444468] Zhen W, Mao L, Yuan Z: Analysis of trample disaster and case study – Mihong bridge fatality in China in 2004. Safety Science 2008;46:1255-1270

[ref-2267906475] Takashi Y, Ishiyam S, Yamada Y, Yamauchi H. Medical triage and legal protection in Japan. The Lancet 2002;359:1949 10.1016/S0140-6736(02)08755-X 12057584

[ref-1310471037] Burkle FM Jr, Hsu EB. Ram Janki Temple: Understanding human stampedes Lancet 2010;377:106-107 10.1016/S0140-6736(10)60442-4 20655584

[ref-2915005722] Sanders AB, Criss E, Steckl P. An analysis of medical care at mass gatherings. Ann Emerg Med 1986;15:515-519 10.1016/s0196-0644(86)80984-2 3963529

